# From Mutation to Manifestation: Penetrance in Amyotrophic Lateral Sclerosis

**DOI:** 10.3390/genes17050576

**Published:** 2026-05-18

**Authors:** Elodie Richard, Sally Al-Hajj Vourc’h, Sylviane Marouillat, Stéphane Beltran, Hélène Blasco, Philippe Corcia, Patrick Vourc’h

**Affiliations:** 1 Service de Biochimie et Biologie Moléculaire, CHRU de Tours, 37044 Tours, France; elodie.richard@etu.univ-tours.fr (E.R.);; 2 Centre de Référence SLA et Autres Maladies du Neurone Moteur, CHRU de Tours, 37044 Tours, France; 3 Imaging Brain & Neuropsychiatry IBraiN U1253, INSERM, Université de Tours, 37020 Tours, France

**Keywords:** ALS, genetics, genetic counseling, variable expressivity

## Abstract

Amyotrophic lateral sclerosis (ALS) is an adult-onset neurodegenerative disease characterized by progressive loss of motor neurons in the brain and spinal cord. While most cases are sporadic, around 10% are familial. Recent genetic studies show that many apparently isolated cases carry pathogenic mutations, highlighting the importance of penetrance, the probability that a causal mutation manifests clinically. This review focuses on mutation penetrance in ALS (*C9orf72*, *SOD1*, *TARDBP*, *FUS* genes), its variability across genes, age, and environmental or genetic modifiers, and its implications for genetic counseling. Identification of pathogenic mutations informs the monitoring of relatives and, in some cases, gives access to targeted therapies or clinical trials. Counseling of asymptomatic relatives must consider incomplete penetrance, which can lead to delayed or absent disease manifestation. ALS exists on a clinical and genetic continuum including related disorders, such as frontotemporal dementia, further influencing risk interpretation. Advances in panel, whole-exome and whole-genome sequencing refine our understanding of penetrance and enable precise diagnostics, and potential tailored therapies. Understanding penetrance is therefore essential to translate mutation discovery into informed clinical decisions and genetic counseling in ALS.

## 1. Introduction

Amyotrophic lateral sclerosis (ALS) is a rare neurodegenerative disorder characterized by progressive degeneration of both upper and lower motor neurons. Motor neuron loss leads clinically to progressively worsening muscle weakness, ultimately resulting in generalized paralysis. The disease course is most often determined by respiratory failure, with a median survival after symptom onset generally ranging from three to five years [1]. Epidemiologically, the annual incidence is estimated at approximately 3 cases per 100,000 individuals, while prevalence is between 7–11 cases per 100,000 [2,3].

Recent advances in molecular genetics have profoundly transformed our understanding of ALS. These studies have identified more than thirty ALS-associated genes and have led, in France, to the implementation of systematic molecular diagnostics for affected patients [3,4]. Furthermore, these discoveries have helped elucidate the underlying pathophysiological mechanisms, highlighting the involvement of complex processes such as dysregulation of gene expression, mitochondrial dysfunction, neuroinflammation, and protein aggregation [5,6].

The first causal gene identified in ALS was *SOD1*. In the initial report describing *SOD1* mutations in multiple ALS families, the mutation was associated with high penetrance in autosomal dominant forms after a certain age [7]. Subsequent studies, however, reported reduced penetrance in other families carrying *SOD1* mutations [8]. These observations highlight the fundamental concept of penetrance in genetics, defined as the proportion of individuals carrying a given mutation and who actually express the associated phenotype. Penetrance is classically classified as complete, when all carriers develop the disease, or incomplete, when only a fraction of carriers manifest the pathology. In ALS, penetrance is frequently incomplete and age-dependent, complicating both the interpretation of genetic data and the prediction of individual risk. Thus, penetrance in ALS should be considered as an age-dependent cumulative risk function, reflecting the probability of disease onset over time rather than a fixed proportion.

The question of penetrance is central in ALS due to the considerable variability observed among individuals carrying the same mutation. Some develop an early-onset, rapidly progressive disease, while others remain asymptomatic throughout their lives. This heterogeneity highlights the presence of genetic, epigenetic, and environmental modulators that influence the transition from mutation to clinical manifestation. In this context, a major question arises: why do some mutation carriers develop the disease while others never exhibit symptoms? Understanding the determinants of this variability is crucial both for elucidating pathophysiological mechanisms and for improving genetic counseling, risk stratification, and the development of personalized therapeutic approaches.

The aim of this review is to provide an update on current knowledge of ALS genetics, considering penetrance as a central aspect of interpretation. It revisits the evolution of conceptual frameworks, moving from the traditional dichotomy between sporadic and familial forms to an approach distinguishing hereditary from non-hereditary forms. The main genes implicated, in both adult-onset and early-onset forms, are examined considering their variable degrees of penetrance. Finally, the clinical implications of these findings are discussed, particularly regarding molecular diagnosis and the practice of genetic counseling.

## 2. Sporadic and Familial Forms of Amyotrophic Lateral Sclerosis

ALS has long been considered as a predominantly sporadic adult-onset neurodegenerative disorder, a view that persisted until the work of Mulder and Kurland in 1954 [9], although rare familial cases had been occasionally reported and Charcot originally described the disease as non-hereditary. Traditionally, sporadic forms, which account for approximately 90% of cases, are distinguished from familial forms, defined by the presence of at least two affected individuals within the same family, typically involving first- or second-degree relatives. Familial ALS represents roughly 10% of cases [10,11]. Most familial cases have a genetic origin and are heritable, explaining their familial clustering.

Recent advances in genetics have revealed that a significant proportion of patients initially classified as sporadic are, in fact, carriers of a causal ALS mutation. These cases often remain labeled as sporadic because only one individual in the family is affected, but the absence of clinical manifestations in relatives may reflect incomplete penetrance, limited family information, or previously imprecise diagnoses [12]. Consequently, some apparently sporadic cases are genetically hereditary, suggesting a genetic and clinical continuum between familial and sporadic ALS rather than strict dichotomy.

From a genetic perspective, it is therefore more relevant to distinguish hereditary forms, characterized by an identifiable genetic cause, from non-hereditary forms, regardless of the number of affected family members, as the identification of a causal variant enables genetic counselling and presymptomatic testing, which are not possible in apparently sporadic cases. Hereditary ALS is most commonly transmitted in an autosomal dominant manner, where a mutated allele is sufficient to confer ALS risk, with a 50% risk of transmission to offspring. Penetrance, however, is variable, meaning that some carriers of a pathogenic allele may remain asymptomatic throughout life [4]. This incomplete penetrance may contribute to the occurrence of apparently sporadic cases, even within families with multiple carriers, complicating the interpretation of family history. Clinical expression of hereditary forms is also heterogeneous, varying in age at onset, disease severity, type of manifestation, and rate of progression. ALS can more rarely be inherited in an autosomal recessive manner, particularly in juvenile- or early-onset forms, when pathogenic variants are present on both alleles of the same gene. A well-described example is the homozygous D91A mutation in *SOD1*, which is associated with a slowly progressive, recessive form of ALS [13]. X-linked forms are exceedingly rare but should be considered, particularly in cases of early-onset disease affecting young males, suggesting the involvement of genes located on the X chromosome. It is also important to note that *de novo* mutations have been reported in ALS but appear to be rare overall, accounting for only a small proportion of cases [14].

Incomplete family histories or previous diagnostic limitations may lead to underestimating the hereditary nature of ALS. Careful exploration of family history is therefore crucial, with particular attention to cognitive or behavioral disorders, including frontotemporal dementia (FTD) [15]. This approach is justified by the clinical and genetic overlap between ALS and FTD: the same mutation may manifest as motor, cognitive, or behavioral impairment in different family members. This phenomenon illustrates the ALS–FTD phenotypic continuum, where clinical presentation can vary considerably between individuals while arising from the same genetic cause.

## 3. Genes Involved in Amyotrophic Lateral Sclerosis

### 3.1. C9orf72 Gene (FTD-ALS1)

The *C9orf72* gene, located on chromosome 9 (locus 9p21.2), encodes a cytoplasmic protein involved in regulating endosomal trafficking. A pathogenic hexanucleotide repeat expansion GGGGCC (G4C2), located in intron 1, represents the major genetic factor in ALS, found in 30–50% of familial cases and 4–10% of sporadic cases [16,17]. In unaffected individuals, the number of repeats generally ranges from 2 to 24, whereas in patients, it can exceed 1000 repeats, with a consensus pathogenic threshold set at 30 repeats [18,19]. C9orf72 repeat expansions vary markedly across populations, with the highest frequencies observed in European populations, especially in Northern Europe, and substantially lower prevalence in Asian and other non-European groups.

The full clinical spectrum of ALS, including fasciculations, muscle cramps, and gait disturbances, can be observed in ALS or ALS-FTD associated with *C9orf72* mutations. The relative frequency of spinal versus bulbar onset is variable across studies, with no consistent predominance. Early cognitive deficits may be present in some individuals initially diagnosed with “pure” ALS [17]. A key part of the clinical evaluation is to investigate the association of ALS with a personal or family history of FTD [20].

**Figure 1:**
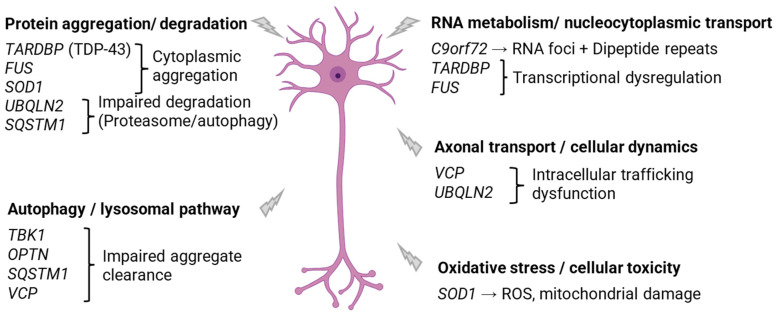
Pathogenic mechanisms of ALS-associated genes. Created with BioRender.com.

The pathophysiological mechanisms underlying *C9orf72* mutations are complex and only partially understood. Functional data support a multiple-hit model combining loss-of-function and toxic gain-of-function mechanisms (Figure 1): On the loss-of-function side, the expanded hexanucleotide repeats lead to reduced expression of the normal C9orf72 protein, which plays a role in endosomal trafficking, autophagy, and immune regulation in neurons and glial cells. Gain-of-function toxicity involves several mechanisms. First, the expanded GGGGCC repeats are transcribed into RNA that forms abnormal structures (RNA foci) that trap RNA-binding proteins, leading to defects in RNA processing, splicing, and nuclear transport. Second, these repeat transcripts undergo translation, producing toxic dipeptide repeat (DPR) proteins. These DPRs accumulate in neurons and glial cells and interfere with key cellular functions, including nucleocytoplasmic transport, protein homeostasis, and ribosomal activity [21].

The study by Douglas et al. [22] estimated the penetrance of the main genes involved in ALS using population-based data from gnomAD and ClinVar. For *C9orf72*, the maximum penetrance, defined as the proportion of expansion carriers who develop ALS or FTD, was estimated at approximately 33%. However, this estimate should be interpreted with caution. Population-based approaches relying on databases such as gnomAD may underestimate true penetrance, particularly due to the age structure of the cohorts. Indeed, a proportion of carriers classified as asymptomatic may actually represent pre-symptomatic individuals who have not yet reached the age at disease onset. Thus, these results reflect an apparent penetrance at the population level but do not fully capture age-related penetrance.

In at-risk families, penetrance is very low before age 40, gradually increases to about 50% by age 60, and is nearly complete by age 80. This indicates that most carriers will develop ALS or FTD during their lifetime, although the age at onset is highly variable [23]. It is, however, difficult to provide a single penetrance figure for the *C9orf72* mutation. Another study emphasizes that *C9orf72* penetrance cannot be described by a single global value; it must be estimated for each family using demographic and pedigree data [24]. This study highlights that penetrance is not uniform across carriers and decreases as the number and age of unaffected at-risk relatives increase. Another interesting study supports the idea that *C9orf72* expansion penetrance is strongly age-dependent but remains variable between individuals, emphasizing the role of modulatory factors in clinical expression [23].

The G4C2 repeat expansion is considered pathogenic when exceeding 30 repeats, and may reach several thousand repeats in some patients. Unlike other repeat expansion disorders, no robust correlation has been demonstrated between the hexanucleotide repeat size of *C9orf72* and penetrance or clinical expression (severity, onset, for example). This lack of correlation may be explained by technical limitations in measuring repeat numbers. The currently favored model is a threshold effect, in which disease risk is associated with the presence of the pathogenic expansion rather than its precise length.

### 3.2. SOD1 Gene (ALS1)

The *SOD1* gene was the first to be implicated in ALS [7] and encodes a homodimeric metalloenzyme, the *SOD1* protein. Pathogenic variants primarily act through a toxic gain-of-function mechanism, promoting protein misfolding and aggregation (Figure 1). In addition, they may contribute to disease pathogenesis through partial loss of function by altering the structure, stability, or protein–protein interactions of *SOD1* [25]. They account for approximately 20% of familial cases in Europe and 2% of sporadic cases [25,26].

More than 180 mutations, predominantly missense and inherited in an autosomal dominant manner (except D91A, D91A/D96N), have been described [27]. They are classically associated with an early age at onset, lower limb onset, and absence of cognitive impairment and a long duration of evolution [28]. The A5V and homozygous D91A variants are well characterized: A5V leads to a pure lower motor neuron involvement with rapid progression (9–13 months), whereas D91A manifests with a prolonged preclinical phase with cramps and dysesthesias, followed by slow progression over several decades. Other variants (H43R, L84V, G86R, N86S, G94A) are associated with survival of less than 3 years, whereas G93C or H46R often allow survival beyond 10 years after symptom onset [29]. Rare cases of rapidly progressive juvenile ALS have also been reported with certain *SOD1* variants [30].

In the original article describing the implication of *SOD1* in ALS, the authors did not explicitly report 100% penetrance of the mutations identified in several families [7]. In 2009, Zinman et al. reported a novel *SOD1* variant associated with familial ALS and reduced penetrance in heterozygous carriers, with disease occurring primarily in the homozygous proband [8]. Here, “low penetrance” refers to the fact that the mutation does not lead to disease in most heterozygous carriers, even though the homozygous proband develops ALS. The proposed explanation is that the mutation, a 6 bp deletion in exon 2, promotes the naturally occurring alternative splicing of exon 2 in *SOD1* mRNA, which in turn lowers the expression of the mutant allele. Consistently, Western blot analysis revealed that carriers of the mutation have markedly reduced levels of *SOD1* protein compared to wild-type individuals or carriers of the A5V *SOD1* mutation. The p.D91A variant of *SOD1* further illustrates this atypical genetic behavior, as it is most commonly associated with autosomal recessive inheritance, where heterozygous carriers are usually unaffected or only rarely develop disease. These observations highlight that *SOD1*-related ALS does not always follow a classical autosomal dominant model, but may instead display variable penetrance or recessive inheritance depending on the variant.

In the study by Douglas et al. [22], the maximum penetrance of *SOD1* was estimated at around 54% in the general population, suggesting incomplete penetrance. For *C9orf72*, similar estimates based on population data may underestimate true penetrance because of age-related bias. However, these estimates hide important variability between *SOD1* variants. Penetrance is not uniform and depends on the specific pathogenic variants, age, and clinical context. Most *SOD1* variants show low penetrance before 40–50 years, with a progressive increase with age. In contrast, common mutations such as A5V, G86R, and G94A are associated with almost complete penetrance in heterozygous carriers after 50–60 years. At the other end, the D91A variant is considered a low-penetrance variant in heterozygous state, with individuals often remaining asymptomatic for many years. Its pathogenic role in this context is still debated. In homozygous state, however, it causes ALS with slow progression.

### 3.3. TARDBP Gene (ALS10, TDP-43)

Mutations in *TARDBP* gene are most often associated with ALS with variable cognitive involvement. The *TARDBP* gene (TAR DNA Binding Protein) encodes the TDP-43 protein, a nuclear protein involved in the regulation of RNA expression, transport, and homeostasis within cells. The frequency of *TARDBP* mutations is approximately 3–5% in familial cases and less than 1% in sporadic cases. These mutations are heterozygous and generally follow an autosomal dominant mode of inheritance [31,32,33].

They are predominantly missense variants, leading to an amino acid change in the C-terminal domain of the TDP-43 protein. Functionally, they promote TDP-43 aggregation in the cytoplasm, resulting in loss of function in the nucleus and cytoplasmic toxicity through sequestration of proteins and RNA (Figure 1). These aggregates are observed in 97% of ALS patients at autopsy, whether in hereditary forms linked to *TARDBP* mutations or in non-hereditary forms [34]. Indeed, even in ALS patients without *TARDBP* mutations, cytoplasmic aggregation of TDP-43 is almost always present, referred to as TDP-43 proteinopathy [35]. This observation suggests that TDP-43 aggregation is a key mechanism in ALS pathophysiology and therefore represents a therapeutic target of interest [36].

For *TARDBP*, Douglas et al. [22] report an estimated penetrance of approximately 38% in the general population. This value should also be interpreted as a population-based estimate, with limitations. In reality, penetrance of *TARDBP* variants is incomplete, age-dependent, and variable depending on the genetic context, with heterogeneous clinical expressivity. Family-based studies have long suggested high penetrance, but this was likely overestimated. The p.M337V and p.A315T variants, commonly used to generate ALS mouse models and relatively frequent in Europe and North America, show high penetrance for p.M337V and intermediate-to-high penetrance for p.A315T.

In contrast, the p.A382T mutation, associated with Sardinia, exhibits reduced-to-moderate penetrance. The reduced penetrance of this variant may be explained by a moderate functional effect on the TDP-43 protein, insufficient on its own to induce motor neuron death. This variant disrupts RNA and protein homeostasis without reaching a pathological threshold. Clinical expression likely depends on additional factors, such as aging or the presence of other genetic risk factors, consistent with a multifactorial model.

### 3.4. FUS Gene (ALS6)

The *FUS* (*FUS*ed in Sarcoma) gene encodes the *FUS* protein, an RNA- and DNA-binding protein that plays a key role in the regulation of RNA expression. Like TDP-43, *FUS* is normally localized in the cell nucleus. Mutations in the *FUS* gene account for 2–4% of familial ALS cases and less than 1% of sporadic cases. These are mainly missense mutations that alter the nuclear localization signal, leading to relocalization and cytoplasmic aggregation of the protein [37,38] (Figure 1). Most mutations are heterozygous and generally follow an autosomal dominant mode of inheritance. *FUS*-related ALS is often characterized by early and cervical motor weakness onset and rapid disease progression. In patients carrying an *FUS* mutation, the mutant protein mislocalizes to the cytoplasm and forms inclusions [39]. Certain *FUS* mutations are more frequently linked to early-onset pure ALS, while other variants can also be associated with the ALS–FTD spectrum. The penetrance of *FUS* mutations is high, with the majority of carriers developing ALS, often before the age of 50, and with rapid disease progression. Some series report that more than 80% of adult carriers develop a clinical phenotype, although phenotypic variability and age at onset may influence this estimate [39,40]. *FUS*-related ALS is characterized by early and aggressive involvement, and cytoplasmic relocalization of *FUS* remains a consistent histopathological marker in affected patients. Notably, *FUS* mutations are also frequently associated with juvenile- or young-onset ALS, often arising from de novo variants, which typically present with a very aggressive disease course and rapid functional decline. These early-onset cases further emphasize the central role of *FUS* in severe, high-penetrance forms of ALS.

It therefore appears, at first sight, surprising that the study by Douglas et al. reports a population-based penetrance estimate for *FUS* of 19% [22]. This discrepancy is likely explained by the rarity of the variants and by ascertainment bias, also known as selection bias, which occurs when the individuals studied are not representative of the general population. In family-based studies, the selection of affected individuals tends to overestimate the true risk, while the rarity of the variants further contributes to this discrepancy.

### 3.5. Other Causal Genes in ALS

In addition to the major genes (*C9orf72*, *SOD1*, *TARDBP*, *FUS*), several other genes have been associated with ALS, although they are much less frequent and generally show incomplete penetrance or penetrance that is difficult to precisely define in available cohorts.

*TBK1* (TANK Binding Kinase 1): The *TBK1* gene encodes a kinase involved in the regulation of autophagy and inflammation. Loss-of-function variants in *TBK1* impair autophagic clearance and promote the accumulation of cytoplasmic aggregates, contributing to the ALS–FTD continuum [41,42]. *TBK1* mutations are observed in <2% of ALS cases and have been described with an autosomal dominant mode of inheritance [43]. Family-based and population-based studies suggest incomplete penetrance, with indicative estimates showing that by around 70 years of age, less than 70% of carriers of pathogenic variants develop ALS, highlighting that many carriers remain asymptomatic even at advanced age.

*VCP* (Valosin Containing Protein): The *VCP* gene encodes an ATPase chaperone essential for protein homeostasis, acting in particular through the ubiquitin–proteasome degradation pathway and autophagy [44]. *VCP* mutations are rare, accounting for less than 1% of ALS cases in European and North American cohorts, and are inherited in an autosomal dominant manner [45]. Clinical manifestations are heterogeneous, including ALS, FTD, and more rarely myopathies, often associated with mislocalization and aggregation of proteins such as TDP-43 or *FUS* in motor neurons.

*OPTN* (Optineurin): The *OPTN* gene encodes an adaptor protein involved in autophagy and mitophagy. Mutations in this gene are rare, accounting for approximately 1% of ALS cases, and contribute to protein accumulation by disrupting interactions with ubiquitin and the clearance of abnormal proteins [42]. Although several heterozygous variants have been described in ALS patients, evidence for high penetrance remains limited, largely based on case reports or small families.

*SQSTM1* (Sequestosome 1, p62): The *SQSTM1* gene encodes the p62 protein, a key player in autophagy and protein homeostasis. Rare variants have been identified in ALS patients, particularly in European familial cohorts; however, *SQSTM1* is not significantly enriched in patients compared to controls. In a study including 486 familial ALS patients, the rare observed variants showed limited segregation and enrichment, suggesting low penetrance [46,47].

*UBQLN2* (Ubiquilin 2): The *UBQLN2* gene encodes a protein that plays a central role in the degradation of ubiquitinated proteins. Mutations in *UBQLN2* cause an X-linked form of ALS, most often dominant, affecting mainly hemizygous males, whereas heterozygous females may show later-onset or variable clinical expression [48]. These mutations are very rare, accounting for less than 1% of ALS cases [49]. The literature suggests that almost all male carriers of pathogenic variants develop the disease between the ages of 30 and 50, whereas disease expression in females may be delayed or partial.

## 4. Genetics of Juvenile Forms of Amyotrophic Lateral Sclerosis

Juvenile ALS, defined by symptom onset before the age of 25, accounts for less than 1% of ALS cases [50]. Compared with adult-onset forms, it more often begins in the lower limbs and generally shows a slower progression, except for certain *FUS* mutations, which can lead to rapid progression even in childhood. These early-onset forms are particularly important to identify in order to guide molecular diagnosis and to organize family management. Several genes have been associated with juvenile forms, with dominant or recessive modes of inheritance and distinct molecular mechanisms.

The *SETX* gene (ALS4, autosomal dominant) encodes senataxin, a helicase involved in RNA metabolism and DNA repair; its mutations can cause dominant juvenile ALS or, in recessive form, progressive ocular motor ataxia (AOA2), illustrating the wide phenotypic spectrum that can arise from a single gene [51]. Autosomal recessive forms include ALS2 (Alsin), involved in endosomal trafficking and motor neuron maintenance, SIGMAR1 (ALS11, sigma-1 receptor), a chaperone protein protecting neurons against endoplasmic reticulum stress, and SPG11 (ALS15, spatacsin), involved in axonal transport and neuronal autophagy [4,52].

In these cases, mutations are generally loss-of-function variants, and the early age at onset is an indirect indicator of often high penetrance, sometimes close to complete penetrance, as observed in recessive forms where homozygous carriers almost invariably develop the disease. These juvenile genes typically lead to progressive motor impairment, sometimes associated with cognitive involvement, in contrast to adult-onset forms with incomplete penetrance, where mutations may remain asymptomatic for decades and clinical expression varies widely depending on age, sex, or genetic context.

## 5. Molecular Mechanisms of Incomplete Penetrance

At the molecular level, variability in the penetrance of a pathogenic mutation in a constitutional genetic disease results from the interaction of multiple mechanisms that modulate the relationship between genotype and phenotype. Among these mechanisms, genetic modifiers play a central role: variants in regulatory genes or cis-regulatory regions can modulate the expression of the mutated gene, attenuating or enhancing the effect of the mutation. These modifiers may, for example, partially compensate for haploinsufficiency or adjust compensatory biological networks, thereby influencing the clinical manifestation of the mutation.

In parallel, stochastic variability in gene expression introduces molecular noise inherent to gene expression processes (transcription and translation). This noise can be amplified by mutations that disrupt regulatory networks, leading to variations in gene expression or protein function that, if they exceed or fall below a certain threshold, determine whether the phenotype emerges [53].

To these factors are added epigenetic and environmental mechanisms, which modify gene activity without altering the DNA sequence and thus contribute to inter-individual variability in penetrance [54]. The combination and interaction of these factors explain why some individuals carrying the same mutation do not develop the disease, or develop it at different ages, illustrating the concept of age-dependent penetrance.

This model is particularly relevant for many neurological diseases, especially ALS, where in certain forms, such as those linked to mutations in the *SOD1* gene, the presence of a mutation does not guarantee immediate disease onset, and where severity and age at onset may vary depending on genetic and environmental context. In SOD1-related ALS, epigenetic and environmental factors do not cause the disease, but they may influence its onset and progression by modulating cellular stress responses, for example. Thus, the interplay of genetic modifiers, expression noise, epigenetics, and environmental factors determines the probability that a carrier of a pathogenic variant will actually develop the disease, explaining the incomplete penetrance observed in many constitutional genetic disorders.

Genetic modifiers that may explain incomplete penetrance in ALS are still relatively few in number. A study by Barbier et al. [55] identified a genetic variant on the X chromosome as a modifier of age at onset in individuals carrying the *C9orf72* G4C2 expansion. This allele is associated with increased expression of SLITRK2, a gene whose protein plays a role at the synapse. The protective allele delays disease onset compared with the deleterious allele, illustrating how variants outside the causal gene can modulate both penetrance and clinical expressivity. This mechanism provides direct evidence of molecular modulation of penetrance in ALS, complementing statistical observations of incomplete penetrance for *C9orf72*, *SOD1*, *TARDBP*, and *FUS*. Another well-described genetic modifier in ALS is the Ataxin-2 (*ATXN2*) gene with intermediate CAG repeat expansions [56]. Intermediate repeat expansions in *ATXN2* have been associated with an increased risk of ALS in carriers of the *C9orf72* G4C2 repeat expansion, suggesting a potential modifier effect on disease susceptibility, although no consistent effect on the development of FTD has been demonstrated.

**Figure 2:**
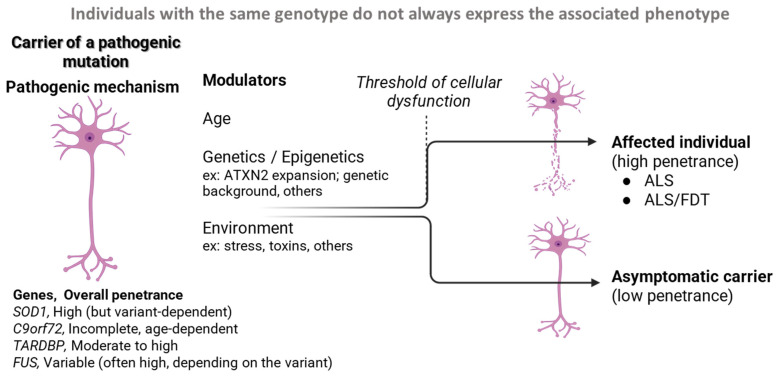
Schematic representation of ALS pathogenesis from pathogenic mutation to clinical phenotype, highlighting the role of various modulators in shaping disease penetrance through modulation of a biological threshold of neuronal dysfunction. Created with BioRender.com.

These observations are consistent with findings from large-scale genetic studies, including those from the Project MinE Consortium, which have highlighted the complex and oligogenic architecture of ALS [57]. Together, they support a model in which rare high-impact variants interact with a broader genetic background to modulate disease risk and penetrance. This model reinforces the need to integrate penetrance into dynamic and individualized risk models, combining genetic background, age, and potential modifiers to improve clinical prediction. Thus, in ALS, the interplay of age, genetic modifiers, epigenetics, and environmental factors determines the probability that a carrier of a pathogenic variant will develop the disease, as these factors converge to influence whether a critical threshold of neuronal dysfunction is reached, thereby determining disease onset (Figure 2).

## 6. Genetic Counselling in Amyotrophic Lateral Sclerosis

Genetic counselling is a process combining medical support and, in some cases, psychological support, aimed at helping patients and their families understand the genetic implications of a disease [58]. In an increasing number of countries, this counselling plays a growing role in the management of ALS. In France, it is offered to all patients, whether in familial or sporadic forms [59]. It is part of a coordinated care pathway, usually within an ALS clinical center or a hospital-based genetic consultation, involving a neurologist, a clinical geneticist, and a genetic counsellor. The identification of a causal mutation in a patient not only confirms the hereditary nature of ALS but also enables presymptomatic testing for at-risk relatives, as well as access to certain targeted therapeutic trials.

A major challenge in genetic counselling in ALS lies in the variable penetrance of mutations. While some mutations in *SOD1* or *FUS* show high, sometimes nearly complete penetrance, others, such as the *C9orf72* expansion or *TARDBP* variants, may remain unexpressed, resulting in asymptomatic carriers even at advanced age. This uncertainty complicates risk assessment for relatives and requires that risk be presented as a probability rather than a certainty, taking into account age, family history, and genetic context.

The literature shows that clinical series are often strongly biased toward symptomatic cases, with little consideration given to asymptomatic carriers in the general population (ascertainment bias). These series therefore do not allow estimation of the proportion of carriers who will never develop ALS. In this regard, population-based penetrance estimates, such as those reported by Douglas et al. [22], are essential. They provide a realistic measure of disease risk for all carriers, independent of multiplex families, and help improve the accuracy of genetic counselling, as well as the planning of clinical trials. An important conclusion of the Douglas et al. study, focusing on the *C9orf72* G4C2 expansion, is that family history remains the best predictor of penetrance in expansion carriers, and that calculating family-specific penetrance allows for more reliable individualized risk estimates.

For genes other than *C9orf72*, *SOD1*, *TARDBP*, and *FUS*, their contribution to ALS is rare, with each gene typically accounting for less than 1% of cases individually. The penetrance of these genes remains difficult to define, in contrast to *SOD1* or certain *TARDBP* variants where data are more robust. For *TBK1*, penetrance is estimated at less than 70% at advanced age, whereas for *VCP*, *OPTN*, *SQSTM1*, and *NEK1*, it appears moderate or variable, consistent with an oligogenic or multifactorial model in which the mutation increases risk but is not sufficient on its own to cause disease. Most available figures reflect frequencies observed in patients compared with controls and serve as indirect indicators of variant impact. They do not allow estimation of age-specific penetrance in the general population, highlighting the need for cautious and nuanced communication in ALS genetic counselling.

## 7. Conclusions

Recent advances in genetics are profoundly transforming the understanding and management of ALS. High-throughput sequencing technologies now allow rapid analysis of gene panels, whole exomes, or even whole genomes, facilitating the identification of pathogenic or likely pathogenic mutations requiring specific follow-up. Recent progress in the genetics of ALS has led to the emergence of gene-targeted therapies, particularly for mutations in *SOD1*, *C9orf72*, *TARDBP*, *and FUS*. Although still largely experimental, these approaches open the way to more targeted and personalized treatment strategies in genetically defined forms of ALS.

Beyond mutation identification, understanding their penetrance is a major challenge: determining why some mutation carriers develop the disease while others do not requires identifying genetic or environmental modifying factors, as well as developing biomarkers capable of predicting conversion to ALS. These data will make it possible to build personalized risk models and better characterize the inter-individual variability of ALS. By combining genetics, penetrance-modulating factors, and predictive biomarkers, it will become possible to anticipate individual risk, personalize follow-up, and develop therapeutic strategies tailored to each patient. To conclude, penetrance is no longer only a theoretical concept used to describe a disease such as ALS; it now helps to interpret individual situations in the context of genetic counselling and to guide concrete medical decision-making.

## Data Availability

No new data were created or analyzed in this study. Data sharing is not applicable to this article.
